# Content and Functionality of United States Medical School Websites

**DOI:** 10.7759/cureus.15534

**Published:** 2021-06-08

**Authors:** Jordan R Pollock, Michael L Moore, Jacob F Smith, Jonny B Woolstenhulme, Dane J Markham, Joshua R Rhees, Kenneth Poole, Nathan T Pollock, Naresh P Patel

**Affiliations:** 1 Orthopedic Surgery, Mayo Clinic Alix School of Medicine, Scottsdale, USA; 2 Dermatology, University of Utah School of Medicine, Salt Lake City, USA; 3 Otolaryngology, Mayo Clinic Alix School of Medicine, Scottsdale, USA; 4 Life Sciences, Brigham Young University, Provo, USA; 5 Internal Medicine, Mayo Clinic Hospital, Phoenix, USA; 6 Neurosurgery, Mayo Clinic Hospital, Phoenix, USA

**Keywords:** medical school, medical school education, medical school wesbite, medical school website content, medical school application, medical school applicant, covid-19, online interview, medical school interview

## Abstract

Introduction

Most medical school applicants use the internet as a source of information when applying for medical school. Previous analyses have evaluated residency and fellowship websites; however, an in-depth analysis of medical school websites is lacking.

Methods

We evaluated 192 United States (US) medical school websites for presence or absence of 39 items relevant to medical school applicants. Items fell into seven general categories: curriculum, research, demographics of incoming class, admissions information, faculty, financial aid, and social.

Results

Of the 192 websites evaluated (152 allopathic and 40 osteopathic schools), websites contained a mean of 23 items (59%) with a standard deviation of 4.2 items.

Conclusion

Our study examining US medical school websites revealed a lack of online information for medical school applicants. As medical school interviews transition to being online during the COVID-19 pandemic, the importance of the medical school website to applicants becomes increasingly crucial. The information contained in our study could be used to improve the functionality and quality of information on medical school websites, which could help both applicants and the medical schools themselves.

## Introduction

Medical school is highly competitive. The acceptance rate of admissions was 41.9% from 2017 through 2020, with an increasing number of applicants each year - 44,869, 51,067, and 52,326 applicants in 2017, 2018, and 2019, respectively [1]. Historically, prospective medical students obtained information about different medical schools through the Medical School Admissions Requirement (MSAR) paper book, summer programs, school advisors, and fairs sponsored by undergraduate institutions. Recently, however, the Association of American Medical Colleges (AAMC) suggests looking at each school's website for program information [2]. In May 2020, a group of national medical education organizations named the Coalition for Physician Accountability made specific recommendations to medical schools and residencies due to the COVID-19 pandemic. These recommendations are predicted to affect at least the 2020 through 2021 application cycle and will significantly limit opportunities for personal interactions between medical school applicants and medical schools [3]. Accordingly, website development, content, and functionality are increasingly important for medical schools. Prior studies have evaluated these factors among residency and fellowship programs, resulting in various recommendations for areas of improvement among their respective program websites to both help applicants and increase recruitment [4-10].

Comprehensive websites can help applicants make informed decisions, providing them with access to information they deem important. For the application cycle of 2019 to 2020, medical school applicants applied to an average of 17 medical schools according to the data recently released by the Association of American Medical Colleges [1]. Medical school applications and interviews are costly for both applicants and programs. Providing applicants with more information to guide decisions regarding which programs to apply to and interview at stands to benefit both parties, especially if it results in better matching of applicants likely to fit a school. Medical schools also stand to benefit from the better matching of applicants, as many institutions fill residency positions with recent graduates from their own programs. The astounding number of applications could be due to the increasingly competitive nature of the medical school or stress and concern for medical school acceptance. These factors may be accentuated during the COVID-19 pandemic as the Medical College Admissions Test (MCAT) has become delayed, and many service and shadowing opportunities have been canceled [11]. 

As medical school applicants apply to, interview at, and ultimately decide which institution to attend, careful planning and research is essential. The internet is easily accessible and multiple studies have shown the importance of websites in recruitment for residency, which likely applies to students applying for medical school as well [5, 8-9]. Medical schools spend substantial time and effort recruiting competitive and diverse students throughout the year with advertisements, information sessions, carefully planned interview days, second-look weekends, phone calls, financial aid offers, and more [12]. While these efforts will continue to be important, maintaining a medical school website with adequate information and quality is paramount, particularly for today’s prospective students. For example, a survey of medical school students applying for residency found that 41% of applicants decided not to apply to at least one program solely based on the quality of its residency website, and 78% of applicants claimed information provided in the residency program website influenced their decision to apply to a particular program [13].

Information sources, such as the MSAR online database and the American Osteopathic Association (AOA) website, were designed to assist medical school applicants while applying for medical school [14-15]. Given the growing reliance on technology to do personal research on different medical schools, the need for prospective medical students to find robust, consistent information online will continue to increase. The main purpose of our study is to provide an in-depth analysis of medical school website content and to assess the functionality of MSAR and AOA for prospective medical school applicants. To our knowledge, this study is the first to do an in-depth analysis of medical school websites in the United States.

This paper was previously posted on the Research Square preprint server on January 22, 2021 (doi: 10.21203/rs.3.rs-147236/v1; http://www.researchsquare.com/article/rs-147236/v1). 

## Materials and methods

The methods of our study were adapted from a well-conceived study examining otolaryngology residency website content [5]. Our study was exempt from Institutional Review Board (IRB) approval because it involves publicly available information. A list of 192 medical school names and website links were obtained from Medical School and Admissions Requirements (MSAR) online database and the American Osteopathic Association (AOA) website in May 2020 [14-15]. We evaluated both information sources for functionality by determining whether the link provided on MSAR or AOA led directly to the medical school homepage or required multiple clicks to get to the medical school homepage. When a link to a program was not available on MSAR or AOA website, we performed a Google search to find the program website. Medical schools without a functional website, or a website that could not be found, were excluded (n = 2).

The data were collected by four authors (JW, JS, DM, JR) between May 2020 and June 2020. Data gatherers searched the websites of these programs for 39 items listed in Table 1. These items were later divided into seven categories for further analysis: curriculum, research, demographics of the incoming class, admissions information, faculty, financial aid, and social. The items and categories included in our study are based on variables many applicants deem as necessary or desirable information, as well as variables we believe are important to medical school applicants [13]. We also included items from a variety of studies examining the quality of residency website content and based our seven categories on these studies [4-6, 9-10]. Variables were also added to our study based on the discretion of the authors of this study, including pre-medical students, medical students, and physicians. Some of these factors on medical school websites could be more important to applicants than other factors, as suggested by studies examining how medical students choose a residency program [16-17]. However, we controlled for this by examining a large number of items (39) on each website and based these items on a variety of studies, as previously described.

**Table 1:** A Comparison of Variables Found on US MD and US DO Medical School Websites COMLEX: Comprehensive Osteopathic Medical Licensing Examination; DO: Doctor of Osteopathic Medicine; GPA: grade point average; Info: information; MCAT: Medical College Admission Test; MD: Doctor of Medicine; Pre-Med: Pre-Medical; Rec: recommendation; stats: statistics; US: United States; USMLE - United States Medical Licensing Examination

Comparison Variables	Number of MD Programs (% of all MD Programs)	Number of DO Programs (% of all DO Programs)	Number of MD + DO Programs (% of all MD and DO Programs)	P-value
Curriculum				
Yearly Overview Listed	146 (96.1%)	40 (100.0%)	186 (96.9%)	0.471
Attendance Policy Listed	42 (27.6%)	10 (25.0%)	52 (27.1%)	0.973
Evaluation (Grading) Policy Listed	57 (37.5%)	8 (20.0%)	65 (33.9%)	0.147
Dual Degree Programs Listed	127 (83.6%)	21 (52.5%)	148 (77.1%)	0.108
Match Results Listed	103 (67.8%)	29 (72.5%)	132 (68.8%)	0.458
Average USMLE Step 1 Score	13 (8.6%)	0 (0.0%)	13 (6.8%)	0.077
USMLE Step 1 Pass rate	29 (19.1%)	1 (2.5%)	30 (15.6%)	0.026
Average COMLEX Score	-	4 (10.0%)	4 (10.0%)	-
COMLEX Pass Rate Listed	-	34 (85.0%)	34 (85.0%)	-
Facility Description	75 (49.3%)	30 (75.0%)	105 (54.7%)	0.018
Rotation Information Provided	109 (71.7%)	32 (80.0%)	141 (73.4%)	0.325
Research				
Description of Research Opportunities	127 (83.6%)	32 (80.0%)	159 (82.8%)	0.821
Research Requirement Information Listed	45 (29.6%)	3 (7.5%)	48 (25.0%)	0.021
Demographics of Incoming Class				
Demographics are Listed	97 (63.8%)	20 (50.0%)	117 (60.9%)	0.526
MCAT Stats Listed	111 (73.0%)	29 (72.5%)	140 (72.9%)	0.698
GPA Stats Listed	115 (75.7%)	26 (65.0%)	141 (73.4%)	0.770
State-by-State Data Listed	27 (17.8%)	9 (22.5%)	36 (18.8%)	0.397
Financial Aid				
Average Financial Aid Listed	7 (4.6%)	0 (0.0%)	7 (3.6%)	0.194
Info on How to Apply For Aid Listed	140 (92.1%)	39 (97.5%)	179 (93.2%)	0.422
Financial Aid Office Contact Info Listed	135 (88.8%)	39 (97.5%)	174 (90.6%)	0.317
Scholarship Opportunities Listed	99 (65.1%)	37 (92.5%)	136 (70.8%)	0.022
Example Budget Listed	137 (90.1%)	33 (82.5%)	170 (88.5%)	0.999
Admissions Info				
Required Pre-Med Courses Listed	145 (95.4%)	40 (100.0%)	185 (96.4%)	0.448
Letters of Rec Requirements Listed	141 (92.8%)	37 (92.5%)	178 (92.7%)	0.644
Admissions Office Contact Info Listed	144 (94.7%)	38 (95.0%)	182 (94.8%)	0.618
Waitlist Information Provided	55 (36.2%)	5 (12.5%)	60 (31.3%)	0.030
Early Decision Program Info Provided	89 (58.6%)	7 (17.5%)	96 (50.0%)	0.003
Acceptance Rate Listed	79 (52.0%)	14 (35.0%)	93 (48.4%)	0.288
In vs Out-of-State Preference listed	59 (38.8%)	12 (30.0%)	71 (37.0%)	0.592
Interview/Decision Timing Listed	115 (75.7%)	17 (42.5%)	132 (68.8%)	0.058
US News Ranking Listed	28 (18.4%)	2 (5.0%)	30 (15.6%)	0.078
Social Environment				
Student Wellness Resources Listed	136 (89.5%)	36 (90.0%)	172 (89.6%)	0.615
Med School Social Media Link(s) Listed	129 (84.9%)	33 (82.5%)	162 (84.4%)	0.759
Description of Area Activities/Highlights Listed	106 (69.7%)	28 (70.0%)	134 (69.8%)	0.665
Clubs/Interest Groups Listed	122 (80.3%)	33 (82.5%)	155 (80.7%)	0.555
Faculty				
Faculty Listed	131 (86.2%)	38 (95.0%)	169 (88.0%)	0.313
Photos Listed	112 (73.7%)	32 (80.0%)	144 (75.0%)	0.394
Student/Faculty Ratio Listed	33 (21.7%)	4 (10.0%)	37 (19.3%)	0.186
Faculty Research Interests Listed	109 (71.7%)	23 (57.5%)	132 (68.8%)	0.563

As the data contained in residency websites can be subjective, we created a standardized process to evaluate the websites, similar to the previous studies in other specialties [4-8]. First, we only searched for the presence or absence of items, with no attempt made to grade the quality or accuracy of the content. Second, any information that was not directly listed on the medical school website was excluded, such as links to external materials or websites, which usually contained general, non-specific information rather than medical school-specific information. Lastly, data were gathered independently by four authors for the same 10 programs and compared for agreement. All authors went through all items again together, noting where a disagreement occurred. Ambiguity in exclusion and inclusion criteria was resolved and adjusted accordingly upon agreement by all authors. After this instruction, data collectors independently gathered the data for all websites (JW, JS, DM, JR, LM). Each website was reviewed by two authors independently, and a third author resolved the disagreement. We performed a descriptive analysis of the data, including means and standard deviations. Additionally, a sub-analysis was performed to determine whether a difference existed in website quality and functionality among the top 25 medical schools and the other 167 schools in our study. We referred to the 2021 data from the US News and World Report website for best medical schools for research, which attempts to rank medical schools based on a variety of criteria [18]. Microsoft® Excel® was used for statistical analysis (Microsoft® Corp., Redmond, WA).

## Results

Of the 192 websites evaluated (152 allopathic and 40 osteopathic schools), websites contained a mean of 23 items (59%) with a standard deviation of 4.2 items. We found a statistically significant difference between allopathic and osteopathic programs (p < 0.05) for six of the 39 variables included in our study. Of these six variables, Doctor of Osteopathy (DO) programs included facility description and scholarship opportunities more frequently than Doctor of Medicine (MD) schools, while MD programs included United States Medical Licensing Examination (USMLE) Step 1 pass rate, research requirement information listed, waitlist information provided, and early decision information provided more frequently. The variables contained least frequently among all medical schools were average financial aid amount (3.6%), average USMLE Step 1 score (6.8%), USMLE Step 1 pass rate (15.6%), and US News and World Report ranking (15.6%). The variables contained most frequently were yearly overview (96.9%), required pre-medical courses (96.4%), admissions office contact information (94.8%), and information on how to apply for financial aid (93.2%) (Table 1).

**Figure 1:**
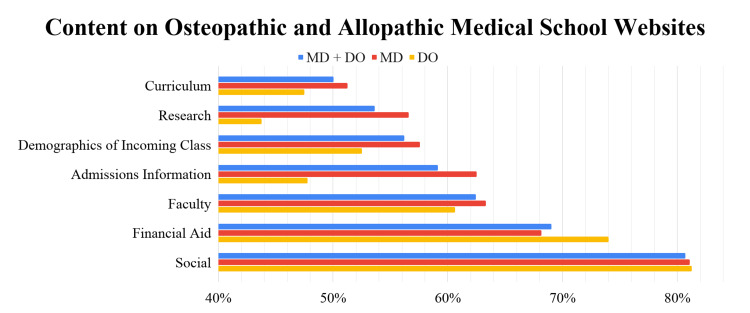
Content analysis comparing osteopathic and allopathic medical school websites DO: Doctor of Osteopathic Medicine; MD: Doctor of Medicine

The categories with the most amount of information across both allopathic and osteopathic schools were the social and financial aid categories, with 81% and 69% of the websites containing this information, respectively. The categories with the least number of variables were the curriculum and research categories at 50% and 54%, respectively. Allopathic websites were more likely to contain variables relating to curriculum, research, demographics, admissions information, and faculty, while osteopathic websites were more likely to contain information in the financial aid and social categories (Figure 1).

**Figure 2:**
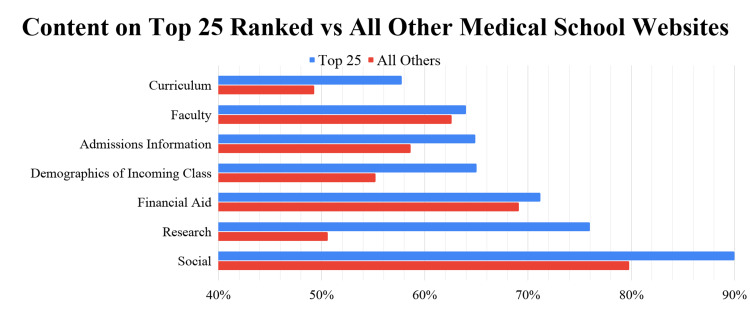
A comparison of variables found on top 25 medical school websites compared to other non-top 25 school websites

Medical school websites from US News and World Report top 25 schools contained more of the 39 variables than schools from non-top 25 programs, with statistical significance demonstrated with variables, such as research requirement, average financial aid, in-state vs out-of-state preference listed, and US News and World Report ranking listed (Table 2) [18]. Medical school websites from top 25 schools had more variables listed on average in each of the seven general categories when compared to the other non-top 25 programs (Figure 2).

**Table 2:** A Comparison of Variables Found on Top 25 Medical School Websites Compared to Other Non-Top 25 School Websites COMLEX: Comprehensive Osteopathic Medical Licensing Examination; GPA: grade point average; Info: information; MCAT: Medical College Admission Test; Pre-Med: Pre-medical; Rec: recommendation; Stats: statistics; US: United States; USMLE - United States Medical Licensing Examination

Comparison Variables	Number of Top 25 Programs (% of all Top 25 Programs)	Number of Non-Top 25 Programs (% of all Non-Top 25 Programs)	P-value
Curriculum			
Yearly Overview Listed	25 (100.0%)	161 (96.4%)	0.702
Attendance Policy Listed	12 (48.0%)	40 (24.0%)	0.076
Evaluation (Grading) Policy Listed	13 (52.0%)	52 (31.1%)	0.201
Dual Degree Programs Listed	25 (100.0%)	123 (73.7%)	0.394
Match results listed	17 (68.0%)	115 (68.9%)	0.612
Average USMLE Step 1 Score	1 (4.0%)	12 (7.2%)	0.489
USMLE Step 1 Pass Rate	3 (12.0%)	27 (16.2%)	0.490
Average COMLEX Score	-	4 (10.0%)	-
COMLEX Pass Rate Listed	-	34 (85.0%)	-
Facility Description	12 (48.0%)	93 (55.7%)	0.382
Rotation Information Provided	22 (88.0%)	119 (71.3%)	0.691
Research			
Description of Research Opportunities	21 (84.0%)	138 (82.6%)	0.661
Research Requirement Information Listed	17 (68.0%)	31 (18.6%)	0.000
Demographics of Incoming Class			
Demographics are Listed	21 (84.0%)	96 (57.5%)	0.278
MCAT Stats Listed	18 (72.0%)	122 (73.1%)	0.597
GPA Stats Listed	19 (76.0%)	122 (73.1%)	0.747
State-by-State Data Listed	7 (28.0%)	29 (17.4%)	0.392
Financial Aid			
Average Financial Aid Listed	4 (16.0%)	3 (1.8%)	0.001
Info on How to Apply For Aid Listed	23 (92.0%)	156 (93.4%)	0.548
Financial Aid Office Contact Info Listed	23 (92.0%)	151 (90.4%)	0.650
Scholarship Opportunities Listed	16 (64.0%)	120 (71.9%)	0.377
Example Budget Listed	23 (92.0%)	147 (88.0%)	0.739
Admissions Info			
Required Pre-Med Courses Listed	23 (92.0%)	162 (97.0%)	0.440
Letters of Rec Requirements Listed	25 (100.0%)	153 (91.6%)	0.884
Admissions Office Contact Info listed	25 (100.0%)	157 (94.0%)	0.790
Waitlist Information Provided	7 (28.0%)	53 (31.7%)	0.543
Early Decision Program Info Provided	13 (52.0%)	83 (49.7%)	0.805
Acceptance Rate Listed	14 (56.0%)	79 (47.3%)	0.864
In vs Out-of-State Preference listed	4 (16.0%)	67 (40.1%)	0.035
Interview/Decision Timing Listed	23 (92.0%)	109 (65.3%)	0.327
US News Ranking Listed	12 (48.0%)	18 (10.8%)	0.000
Social			
Student Wellness Resources Listed	22 (88.0%)	150 (89.8%)	0.541
Med School Social Media link(s) Listed	23 (92.0%)	139 (83.2%)	0.933
Description of Area Activities/Highlights Listed	22 (88.0%)	112 (67.1%)	0.512
Clubs/Interest Groups Listed	23 (92.0%)	132 (79.0%)	0.884
Faculty			
Faculty Listed	20 (80.0%)	149 (89.2%)	0.337
Photos Listed	18 (72.0%)	126 (75.4%)	0.510
Student/Faculty Ratio Listed	8 (32.0%)	29 (17.4%)	0.213
Faculty Research Interests Listed	18 (72.0%)	114 (68.3%)	0.796

Lastly, in terms of website accessibility, 89% of medical school program listings on the MSAR database or the AOA website provided direct links, 10% provided absent or non-functional links, and 1% provided indirect links.

## Discussion

A recent study examining prospective students’ medical school preferences reported the most important factors for medical school choice were academic prestige, location, and the “intangibles,” such as “gut feelings” and personal interactions [19]. According to our study, medical schools rarely included the US News and World Report rankings on their websites, average USMLE Step 1 score, or USMLE Step 1 pass/fail rate. These factors, which are associated with the prestige of the school, should be incorporated into medical school websites. With regards to location, medical schools provided descriptions of the location of the medical school nearly 70% of the time. This becomes increasingly important with online interviews as many applicants may not be able to see the area for themselves. Another area for improvement for medical school websites could be providing state-by-state demographic information, which was only listed 19% of the time. However, the “intangibles,” such as “gut feelings” and personal interactions, were student wellness resources listed, clubs and interest groups, and social media links which were listed 90%, 81%, and 84% of the time, respectively. This shows medical schools are likely aware of the “intangibles” and attempt to address them with more personal and social content.

With the rise of social media for recreational and professional purposes, integrating social media effectively and efficiently could help medical schools recruit desired applicants and help applicants learn more about different schools. We found 84% of medical schools’ websites contained links to a form of social media representing their program, but 16% of programs did not have a directly accessible social media page for their program. This suggests an area for improvement. Having a social media site available for applicants could prove useful to students and programs, as a study done involving nearly 1,000 medical students applying for residency showed that 68% of students reported using social media to learn about programs and 10% reported that the information found in the social media pages influenced their decisions on where to apply [20]. Similarly, a survey of medical students applying for residency suggested social media as an efficient method for highlighting social activities to improve recruitment [21].

Information on current enrolled medical students, such as class demographics and state-by-state data, may be the only exposure of such applicants to the unique personalities and backgrounds of students in the program before deciding to apply to a program. Class demographics and state-by-state data were only listed on 61% and 19% of medical school websites, respectively. Medical schools may benefit from improving these sections of their websites. We also found medical school websites do not have much information pertaining to individual students. However, many residency websites often include photos of the class, photos of each individual student, and small, personal descriptions of each resident. This is an area for medical school websites to give more personalization to their program. Of course, maintaining the appropriate confidentiality of the students should be considered.

Additionally, we found curriculum and research were not adequately addressed on medical school websites. For example, attendance policy was only listed on 27% of websites, grading policies were listed on 34% of websites, and research requirement information was listed on 25% of websites. As medical education becomes more personalized, descriptive details of programs could help students choose a school based on their unique learning styles [22-24]. These details are essential as applicants choose to which programs they will apply. Also, while many of these topics are discussed during interviews, these details can be forgotten or unclear, and a more robust website would be useful in addressing these important questions when medical students are deciding which school to attend. Including more information on curriculum and research could help medical students decide which programs to apply to and attend and help programs recruit students who are a better fit for the curriculum of the school. Recent articles have suggested that residency programs expand the amount of information for applicants during the COVID-19 interview cycle, and perhaps the same should apply to medical schools [25].

While some aspects of medical school websites are lacking, we found medical school website links to be functional through the MSAR database and the AOA website. Of the 192 medical schools examined in our study, 89% of medical school program listings in AOA or MSAR provided direct links, while only 10% were absent or non-functional and 1% were indirect links. However, the number of multiple-step, absent, and non-functional links could still be improved.

The top 25 schools in the US, according to the US News and World Report 2021, had more study variables listed on their websites than non-top 25 schools, such as information on the research requirement and average financial aid. In addition, allopathic websites were more likely to contain variables relating to curriculum, research, demographics, admissions information, and faculty, while osteopathic websites were more likely to contain information in the financial aid and social categories [18]. Future investigations should determine why these differences exist, and whether these differences affect prospective student recruitment. Future research should aim to determine how the COVID-19 pandemic and lack of online information on medical school websites could be affecting the number of applications submitted per applicant.

Limitations of our study include the subjective nature of analyzing medical school websites. However, we feel our method of data collection was standardized sufficiently to control for ambiguity. Another limitation was the lack of established standardized criteria for evaluating websites. Every item that could be important to a medical school applicant was not analyzed. As a solution, we included a variety of items and developed search criteria based on studies evaluating residency website content and the recent experience of the authors of our study. Lastly, only including items listed directly on the medical school website rather than on external links could underestimate the presence of items on websites in our study. However, this was an important factor to determine the accessibility and functionality of information and the user-friendly status of the websites. Our study does not address the accuracy or quality of information contained on websites. Notwithstanding these limitations, we believe our analysis provides valuable insight for medical school directors, website developers, and medical school applicants. Future areas of study could include an in-depth analysis of social media use among medical schools, how website quality affects the number of medical school applications, and why differences in website content exist between different programs, such as allopathic, osteopathic, and the US News and World Report top 25 schools.

## Conclusions

The 2020-21 residency application cycle poses a new challenge for applicants and programs. As most interviews are being conducted on a virtual platform, interview costs are less of an issue for applicants, which could drive up the number of programs that students apply to and consider. A robust source of information available to applicants on medical school websites serves to benefit both the applicant and program alike to prevent overwhelming medical schools with applications and interviewees.

Medical school website quality is important to medical school applicants, and our study identifies several areas where programs could focus efforts for website renovation. The categories of variables which are included least frequently on medical school websites are variables related to curriculum, research, demographics, and admissions information. The results from our study can be used to improve medical school websites to the benefit of applicants and medical schools.
